# Behavioral Outcomes Following Brain–Computer Interface Intervention for Upper Extremity Rehabilitation in Stroke: A Randomized Controlled Trial

**DOI:** 10.3389/fnins.2018.00752

**Published:** 2018-11-08

**Authors:** Alexander B. Remsik, Keith Dodd, Leroy Williams, Jaclyn Thoma, Tyler Jacobson, Janerra D. Allen, Hemali Advani, Rosaleena Mohanty, Matt McMillan, Shruti Rajan, Matt Walczak, Brittany M. Young, Zack Nigogosyan, Cameron A. Rivera, Mohsen Mazrooyisebdani, Neelima Tellapragada, Leo M. Walton, Klevest Gjini, Peter L.E. van Kan, Theresa J. Kang, Justin A. Sattin, Veena A. Nair, Dorothy Farrar Edwards, Justin C. Williams, Vivek Prabhakaran

**Affiliations:** ^1^Department of Radiology, University of Wisconsin – Madison, Madison, WI, United States; ^2^Department of Kinesiology, University of Wisconsin – Madison, Madison, WI, United States; ^3^Institute for Clinical and Translational Research, University of Wisconsin – Madison, Madison, WI, United States; ^4^Department of Biomedical Engineering, University of Wisconsin – Madison, Madison, WI, United States; ^5^Department of Educational Psychology, University of Wisconsin – Madison, Madison, WI, United States; ^6^Center for Women’s Health Research, University of Wisconsin – Madison, Madison, WI, United States; ^7^Neuroscience Training Program, University of Wisconsin School of Medicine and Public Health, Madison, WI, United States; ^8^Department of Materials Science and Engineering, University of Wisconsin – Madison, Madison, WI, United States; ^9^Department of Electrical and Computer Engineering, University of Wisconsin – Madison, Madison, WI, United States; ^10^Department of Psychology, University of Wisconsin – Madison, Madison, WI, United States; ^11^Clinical Neuroengineering Training Program, University of Wisconsin – Madison, Madison, WI, United States; ^12^Medical Scientist Training Program, University of Wisconsin School of Medicine and Public Health, Madison, WI, United States; ^13^Department of Neurology, University of Wisconsin – Madison, Madison, WI, United States; ^14^Department of Neurological Surgery, University of Wisconsin – Madison, Madison, WI, United States; ^15^Department of Psychiatry, University of Wisconsin – Madison, Madison, WI, United States

**Keywords:** brain–computer interface (BCI), stroke, recovery, rehabilitation, motor function, hemiparesis, upper extremity

## Abstract

Stroke is a leading cause of persistent upper extremity (UE) motor disability in adults. Brain–computer interface (BCI) intervention has demonstrated potential as a motor rehabilitation strategy for stroke survivors. This sub-analysis of ongoing clinical trial (NCT02098265) examines rehabilitative efficacy of this BCI design and seeks to identify stroke participant characteristics associated with behavioral improvement. Stroke participants (*n* = 21) with UE impairment were assessed using Action Research Arm Test (ARAT) and measures of function. Nine participants completed three assessments during the experimental BCI intervention period and at 1-month follow-up. Twelve other participants first completed three assessments over a parallel time-matched control period and then crossed over into the BCI intervention condition 1-month later. Participants who realized positive change (≥1 point) in total ARAT performance of the stroke affected UE between the first and third assessments of the intervention period were dichotomized as “responders” (<1 = “non-responders”) and similarly analyzed. Of the 14 participants with room for ARAT improvement, 64% (9/14) showed some positive change at completion and approximately 43% (6/14) of the participants had changes of minimal detectable change (MDC = 3 pts) or minimally clinical important difference (MCID = 5.7 points). Participants with room for improvement in the primary outcome measure made significant mean gains in ARAT_total_ score at completion (ΔARAT_total_ = 2, *p* = 0.028) and 1-month follow-up (ΔARAT_total_ = 3.4, *p* = 0.0010), controlling for severity, gender, chronicity, and concordance. Secondary outcome measures, SIS_mobility_, SIS_adl_, SIS_strength_, and 9HPT_affected_, also showed significant improvement over time during intervention. Participants in intervention through follow-up showed a significantly increased improvement rate in SIS_strength_ compared to controls (*p* = 0.0117), controlling for severity, chronicity, gender, as well as the individual effects of time and intervention type. Participants who best responded to BCI intervention, as evaluated by ARAT score improvement, showed significantly increased outcome values through completion and follow-up for SIS_mobility_ (*p* = 0.0002, *p* = 0.002) and SIS_strength_ (*p* = 0.04995, *p* = 0.0483). These findings may suggest possible secondary outcome measure patterns indicative of increased improvement resulting from this BCI intervention regimen as well as demonstrating primary efficacy of this BCI design for treatment of UE impairment in stroke survivors.

**Clinical Trial Registration:** ClinicalTrials.gov, NCT02098265.

## Introduction

### Stroke

Each year there are approximately 800,000 new incidences of stroke in the United States (Benjamin et al., 2017), and in 2010 there were an estimated 16.9 million stroke events globally (Mozaffarian et al., 2015). Stroke occurs as a result of a blockage of blood flow in an area of the brain or by rupture of brain vasculature causing death or damage to local and distal brain tissue. In either etiology, survivors may experience some level of upper extremity (UE) physical impairment. Despite recent advances in acute care, an increasing number of stroke survivors face long-term motor deficits (Benjamin et al., 2017). Costs of care for long-term disability resulting from stroke are substantial with the direct medical costs of stroke estimated to $17.9 billion in 2013 (Benjamin et al., 2017). It is crucial that motor therapy for stroke enhances a survivor’s capacity to autonomously participate in activities of daily living (ADLs), thereby decreasing dependency on caregivers as well as the cost and level of care necessary (Dombovy, 2009; Stinear, 2016). Efficacious motor therapy should be designed to improve the overall quality of life for the individual survivor based on their goals and needs (Remsik et al., 2016; Stinear, 2016).

### Need for Treatment

Survivors in the chronic stage of stroke are the most desperate for rehabilitation. Existing pharmacological treatments and behavioral therapy methods primarily serve to treat symptoms associated with stroke (Benjamin et al., 2017) and may not bring about optimal changes in brain function or connectivity (Power et al., 2011; Nair et al., 2015). While a growing population of research suggests the greatest potential for recovery in the post-stroke brain occurs within the first months after insult (Stinear and Byblow, 2014), neuroplastic capacity has been demonstrated in both acute and chronic phases (Caria et al., 2011; Ang et al., 2015). Spontaneous biological recovery (SBR) (Beebe and Lang, 2009; Cramer and Nudo, 2010) in the initial days and weeks following stoke (acute phase) is thought to represent a critical period in the complex progression of motor recovery, which combines neurobiological processes and learning-related elements. After this window of SBR, it is posited a sensitive period of neurorecovery persists, plateauing around 6 months post-stroke (Wolf et al., 2006, 2010; Dromerick et al., 2009; Cramer and Nudo, 2010). Traditional rehabilitation therapies generally lose efficacy after such time and the course of standard of care treatment options is exhausted leaving chronically impaired persons with few options.

### Potential for Treatment

Motor and cognitive recovery after these initial windows may no longer occur in the same spontaneous nature as is observed during SBR. However, innovative therapeutic techniques show some efficacy generating functional motor recovery beyond the traditional rehabilitation windows (Cramer and Nudo, 2010; Ang et al., 2015; Irimia et al., 2016). Brain–computer interfaces (BCIs), a novel rehabilitation tool, have shown proof of concept for rehabilitating volitional movements in stroke survivors (Muralidharan et al., 2011; Song et al., 2014, 2015; Young et al., 2014a,b,c,d, 2015; Irimia et al., 2016). In this growing area of research, developing technologies demonstrate promising potential for treating hemiparesis in a clinically viable and efficient manner and they may offer an avenue to increased autonomy for patients reducing their cost and burden of care.

### Effectiveness of Current BCI Therapies

There is currently considerable variability in design and efficacy of BCI therapies as well as little consensus with respect to proper arrangement, administration, and dosing (Muralidharan et al., 2011; Ang and Guan, 2013; Young et al., 2014a; Ang et al., 2015; Irimia et al., 2016; Remsik et al., 2016; Bundy et al., 2017; Dodd et al., 2017). Although acute stroke care has improved morbidity outcomes significantly, current treatments for persistent UE motor impairment resulting from stroke offer only limited restoration of UE motor function the further from stroke a survivor progresses (Wolf et al., 2006, 2010; Dromerick et al., 2009; Benjamin et al., 2017; Stinear et al., 2017). Evidence suggests both acute and chronic stroke patients respond to various neuro-rehabilitative BCI therapy strategies and can achieve clinically significant changes in measures of UE impairment (Young et al., 2014c; Irimia et al., 2016; Remsik et al., 2016). Furthermore, recent research also suggests that BCI therapy targeted at motor recovery may provide benefits in other brain regions outside of only the motor network (Mohanty et al., 2018).

### Overview of This Study

This *post hoc* analysis of an ongoing clinical trial (NCT02098265) (Song et al., 2014, 2015; Young et al., 2014a,b,c,d, 2015) evaluates the effects of an interventional, non-invasive closed-loop electroencephalography (EEG)-based BCI intervention for the restoration of distal UE motor function in stroke survivors. Participants who showed measurable change in the primary outcome measure were grouped *post hoc*. This sub-analysis seeks to identify whether there are participant characteristics strongly associated with motor improvement as measured by primary and secondary outcome measures of UE function. These analyses are intended to inform future BCI research approaches and intervention designs as well as suggest and encourage appropriate participant selection.

## Materials and Methods

### Ethics Statement

Participants were recruited as part of an ongoing prospective randomized, cross-over control design stroke rehabilitation study. This study was designed to investigate interventional BCI intervention targeting UE motor function in stroke survivors. This study was approved by the University of Wisconsin Health Sciences Institutional Review Board (Study ID 2015-0469); all subjects provided written informed consent upon enrollment. A CONSORT flow diagram is made available in the Supplementary Material.

### Study Design and Subjects

#### Recruitment and Enrollment

This ongoing study, registered with ClinicalTrials.gov (study ID NCT02098265), utilizes an open call for participants with a wide range of (1) UE hemiparesis resulting from stroke, (2) time-since-stroke, (3) stroke type, (4) lesion location, (5) number of previous stokes, and (6) stroke severity. Subsequent to informed, written consent, stroke survivors were randomized, by permuted-block design accounting specifically for gender, stroke chronicity (<1 year, ≥1 year), and severity of motor impairment (mild, severe) as measured by the Action Research Arm Test (ARAT) (mild = ARAT_total_ of >28, severe = ARAT_total_ ≤ 27) [*n =* 21, mean age = 61.6 years ± 15 years, 10 female, 4 concordant lesions (stroke lesion impairs preferred dominant hand as assessed by the Edinburgh Inventory (Oldfield, 1971), mean chronicity = 1127 ± 1327 days, 12 participants presented with severe UE motor deficit, mean baseline ARAT score of impaired side = 26.6 ± 26.1, Delayed Therapy Group (DTG) *n* = 12, Immediate Therapy Group (ITG) *n* = 9]. Chronicity is measured as time since stroke, in days, to baseline measurement day. Participant characteristics are displayed in Table 1.

**Table 1:** Participant demographics and baseline characteristics.

Participants	Age (years)	Chronicity days	Severity	Clinical cause lesion location	Baseline ARAT	Completion ARAT	Follow up ARAT	ARAT change	FMA-UE change
1	47–51	160	Severe	L-Lateral medulla	3	2	7	–1 (4^∗∗^)	–2 (9^∗∗∗^)
2	49–53	490	Severe	R-MCA stroke	3	4	8	1^∗^ (5^∗∗^)	2^∗^ (11^∗∗∗^)
3	76–80	658	Mild	Leg/periventricular white, MHR	57	57	57	0 (0)	0 (0)
4	67–51	2723	Severe	R-PLIC putamen	23	40	39	17^∗∗∗^ (16^∗∗∗^)	I3^∗∗∗^ (12^∗∗∗^)
5	81–85	580	Mild	Cerebellar vermis	47	52	52	5^∗∗^ (5^∗∗^)	2^∗^ (2^∗^)
6	73–77	197	Severe	R-prefrontal, midfrontal, temporal	0	0	3	0 (3^∗∗^)	0 (7^∗∗∗^)
7	62–66	101	Mild	R-white matter	56	57	57	1^∗^ (1^∗^)	7^∗∗∗^ (7^∗∗∗^)
8	40–44	2645	Severe	R-frontal parietal	7	7	7	0 (0)	0 (0)
9	55–59	588	Severe	R-MCA	3	4	0	1^∗^ (–3)	2^∗^ (–7)
10	45–49	452	Severe	L-hemorrhagic stroke	0	2	0	2^∗^ (0)	4^∗∗^ (0)
11	30–34	494	Mild	L-ICA	57	57	57	0 (0)	0 (0)
12	60–64	44	Mild	L-PCA	57	57	57	0 (0)	0 (0)
13	57–61	849	Mild	L-MCA	57	57	57	0 (0)	0 (0)
14	44–48	3017	Severe	R-MCA/R-FI	3	4	5	1^∗^ (2^∗^)	2^∗^ (4^∗∗^)
15	69–73	790	Severe	R-MCA/R-TP	3	0	3	–3 (0)	–7 (0)
16	78–82	631	Mild	R-Occipital	57	57	57	0 (0)	0 (0)
17	75–79	5125	Severe	R-MCA/ACA	9	11	10	2^∗^ (1^∗^)	4^∗∗^ (2^∗^)
18	42–46	177	Mild	L-MCA	57	57	57	0 (0)	0 (0)
19	62–66	392	Severe	R-frontal hematoma R-VAOA	3	5	16	2^∗^ (13^∗∗∗^)	4^∗^ (29^∗∗∗^)
20	55–59	2767	Mild	Subarachnoid hemorrhage	57	57	57	0 (0)	0 (0)
21	69–73	783	Severe	R-MCA	0	0	0	0 (0)	0 (0)

Mean	61.6	1127			26.6	28.1	26.8	1.3 (2.2)	1.5 (3.6)
(A) Median	61.9	588			9	11	16	0 (0)	0 (0)
SD	15	1327			26.4	26.3	25.9	3.9 (4.5)	3.8 (7.4)

Mean	61.1	1289			11.4	13.4	14.8^∗^	2 (3.4)	2.2 (5.4)
(B) Median	64	584			3	4	7	1 (1.5)	2.0 (3.0)
SD	13.5	1497			18	20.2	19.6	4.7 (5.2)	4.5 (8.5)


*ARAT indicates Action Research Arm Test; FMA-UE indicates fugl-meyer assessment of upper extremity; MCA indicates middle cerebral artery; ICA indicates internal carotid artery; PCA indicates posterior cerebral artery; FI indicates frontoparietal infarct; TP indicates temporalfrontal-parietal; ACA indicates anterior cerebral artery; MHR indicates motor hand region; VAOA indicates vertebral artery origin aneurysm; L, left; R, right. ARAT change: completion-baseline (follow up-baseline). (A) indicates descriptive statistics for all (*n* = 21) participants; (B) indicates descriptive statistics for (*n* = 14) participants able to achieve ARAT improvements (ceilings removed). FMA-UE is a predicted change that was used to approximate equivalent score that assesses the association between the categorical range of ARAT scores, ^∗^ indicates responder (Δ_*ARAT*_ = 1); ^∗∗^ indicates minimal detectable change (MDC) (Δ_*ARAT*_ = 3);^∗∗∗^ indicates minimal clinically important difference (MCID) (Δ_*ARAT*_ = 5.7).*

#### Inclusion–Exclusion Criteria

**FIGURE 1:**
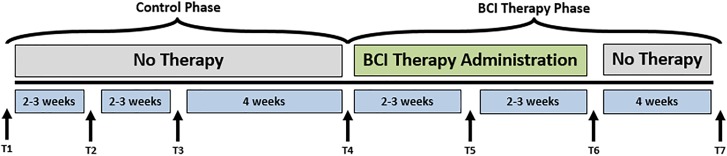
Study design. The time-points at which neuroimaging data were collected are represented by Tl, control baseline 1; T2, control baseline 2; T3, control baseline 3; T4, therapy baseline; T5, mid-therapy; T6, post-therapy; and T7, one-month post-therapy. While the crossover control group (DTG) completed visits T1–T7, the immediate therapy group (ITG) completed visits T4–T7 only.

Potential participants met inclusion criteria if they were aged 18 years or older, had persistent UE motor impairment resulting from stroke, and no other known neurologic, psychiatric, or developmental disabilities. Exclusion criteria were: allergies to electrode gel, surgical tape, and/or metals, concurrent treatment for infectious disease, apparent lesions or inflammation of the oral cavity, pregnancy or intention to become pregnant during the study, and any contraindication for magnetic resonance imaging (MRI). Subjects were excluded from the presented analyses if they (1) failed to complete at least 9 of 15, 2-h BCI intervention sessions occurring at least twice each week, (2) failed to complete all four MRI and behavioral testing sessions occurring in the intervention phase (Figure 1; see Supplementary CONSORT Flow Diagram).

#### Randomization and Study Schema

**FIGURE 2:**
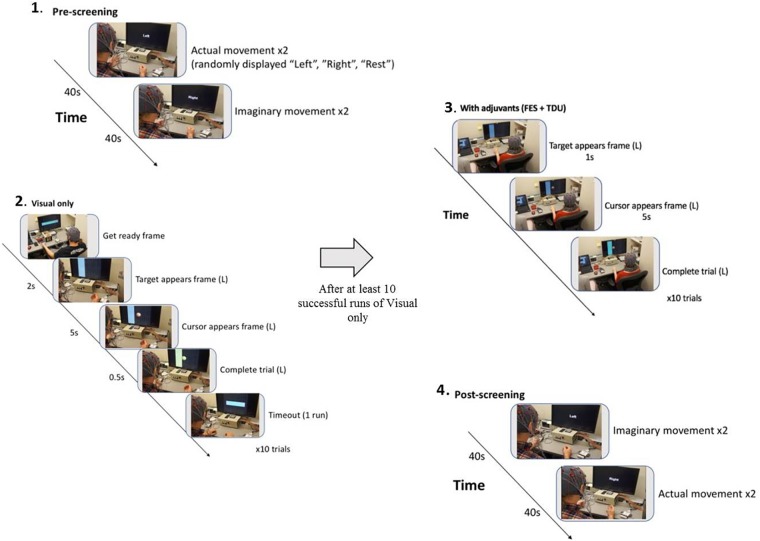
BCI intervention block design: (1**)** A pre-session open-loop screening task of two attempted and then two imagined grasping tasks (left, right, rest) is used to set control features (BCI classifier) for the forthcoming intervention task (Cursor Task). (2) The closed-loop cursor and target (visual only) intervention condition consists of at least 10 runs of 10 trials of attempted grasping movements for the purpose of guiding a virtual cursor (Ball) either left, or right as cued by the target (Goal) presentation on the horizontal edge of the screen. (3) Following 10 successfully completed runs of the visual only condition, adjuvant stimuli are added to enrich the feedback environment and facilitate volitional movement of the affected extremity (grasping). Subsequent runs are attempted at the preferred pace of the participant, completing as many runs as time allows. (4) With 15 min remaining in the 2-h intervention session, the participant is switched into the post-session open-loop screening task of two imagined and then two attempted grasping tasks (left, right, rest).

Participants were randomly assigned to either receive BCI intervention immediately (ITG) following consent or to a DTG wherein participants were neither prohibited continuation of customary care, nor did they receive any BCI intervention. Participants, when receiving the BCI intervention condition, had at least 9 and up to 15 BCI intervention sessions (two-to-three sessions/week) wherein they received BCI intervention (Figure 2) lasting up to 2 h for a potential total dosing of 30 h of BCI intervention. Along with the BCI intervention sessions, subjects also received fMRI and behavioral testing at four-time points: prior to the first BCI intervention session (baseline, T4), after the first few weeks of intervention (midpoint, T5), immediately following the final intervention session (completion, T6), and again 1 month after the endpoint assessment (follow-up, T7) (Figure 1). Later in this publication, the authors will refer to time points 1–4 with the intention of describing time points 1–4 of the intervention phase (T4–7 from Figure 1). Because T1–4 in Figure 1 refer to the control phase, the authors from here forward will refer to any data from these points by explicitly stating when the control phase is being considered.

#### Crossover Design

Following the final testing session, participants in the DTG cross over to the experimental or intervention phase and begin study visits for the BCI intervention condition as illustrated in Figure 1. For participants in the DTG, the crossover time point (T4) represents baseline as it is measured immediately prior to participation in BCI intervention sessions.

### Outcomes

For these sub-analyses, and consistent with original study design, a primary objective outcome measure of UE function, the ARAT (Mathiowetz et al., 1985; Beebe and Lang, 2009; Malhotra et al., 2016), and secondary outcome measures of function (capacity and performance) including the self-report Stroke Impact Scale (SIS) (Duncan et al., 1999; Lin et al., 2010), Hand Grip Strength (An et al., 1980; Malhotra et al., 2016), and the 9-Hole Peg Test (9HPT) (Mathiowetz et al., 1985) were assessed in the 21 participants who met the aforementioned criteria. The primary outcome measure, with registered minimal detectable change (MDC) and minimal clinically important difference (MCID) values (ARAT MDC_90_ ≥ 3 point change, MCID ≥ 5.7 point change) (Lang et al., 2006; Simpson and Eng, 2013), was chosen to obtain clinically reliable measures of UE motor function change as a result of BCI intervention. 9HPT was included in this report as an additional objective (time) measure of motor function. The 9HPT is an assessment of fine motor control and speed of distal UE movement capacity and performance. The 9HPT requires finger dexterity and grip, and supplements the ARAT as they both assess gross UE capacity and function. This study analyzes ARAT scores, 9HPT performance by the affected UE (9HPT_affected_), and SIS sub-scores of the impaired hand from the four time points, illustrated in Figure 1. The Fugl-Meyer Assessment of the Upper Extremity (FMA-UE) is another objective measure of function commonly used to assess UE capacity in several BCI studies. Although the FMA-UE was not intended as an assessment in this design, associations between categorical ranges of ARAT score and FMA-UE score, as presented in Hoonhorst et al. (2015), were used to approximate equivalent FMA-UE scores for the purpose of convenient comparison between the presented ARAT outcome scores and behavioral changes presented in previous publications. ARAT scores within the Upper-Limb category defined by baseline measures (Hoonhorst et al., 2015) were mapped to the FMA-UE score within the same category, rounded to the nearest whole integer, as FMA-UE measurements give scores in integer values.

### Description of the Behavioral Outcome Measures

The primary outcome measure was the ARAT. The ARAT is a 57-point metric capable of assessing specific changes in upper limb function with sub-components for grasp, grip, pinch, and gross motor movement all of which sum to the total ARAT (Hsieh et al., 1998). The secondary outcome measures included the SIS, widely used to measure quality of life in stroke survivors, that consists of eight dimensions and a composite disability score (Vellone et al., 2015). The SIS is a 59-item patient-reported outcome measure, covering eight domains: strength (4 items), hand function (5 items), mobility (9 items), ADLs (10 items), memory (7 items), communication (7 items), emotion (9 items), and handicap (8 items). The domains are scored on a metric of 0–100, with higher scores indicating better self-reported health (Vellone et al., 2015). As it is possible the ARAT does not entirely capture the extent to which participants can functionally interact with their surroundings outside the laboratory, this subjective measure was chosen to support and record the participants’ personal experience and opinion of their functional capacities relative to real-world application (Waddell et al., 2017). Self-report metrics are important for understanding the extent to which a participant is recovering UE motor activities subjectively in a real-world setting (outside the testing room setting) (Stinear et al., 2017). An additional secondary outcome measure was the 9HPT, which is a brief, standardized, quantitative test of UE function (Mathiowetz et al., 1985). The score for the 9HPT is an average of the two trials (Mathiowetz et al., 1985). Finally, a Smedley spring-type dynamometer tested the average grip strengths in pounds (lbs.) over three repeated trials per assessment to measure participant grip strength (An et al., 1980; Malhotra et al., 2016).

### Analysis of Outcome Measures

Data analysis of outcome measures examined four central relationships: (1) Change in outcome measure scores over time (Table 2); (2) primary outcome measure improvement rate differences between intervention and control (Table 3); (3) improvement rate differences in outcome scores between subjects who realized an increase in primary outcome (responders) and non-responders (Table 4); and (4) differences in covariates and outcome measurements between responders and non-responders (Table 4) for the purpose of discerning characteristic trends of those participants who best respond to this BCI intervention. It is important to note that for all responder analyses, participants who scored a perfect 57 total score at baseline and completion were excluded from the sample (*n* = 7 excluded) due to an inability to show improvement in primary outcome leaving *n* = 14 subjects remaining for all the responder sub-analyses. Likelihood ratio tests of linear mixed effect (LME) models offered rigorous analysis for each research question while paired and independent samples *t*-tests provided analysis of more general trends that LME may miss. Testing excluding the follow-up time period (time periods 1–3 of intervention) allowed for examination of direct effects of the BCI intervention while parallel analyses including the follow-up time point (time periods 1–4) gave insight into potential lasting effects of the BCI intervention.

**Table 2:** Summary of outcome measures during assessment and including follow-up of BCI therapy.

Outcome measures	Improvement score Mean ± SD	LME Estimate ± SE	Covariates	*T*-test *p-*Value	Time LME *p-*Value
**Stroke impact scale (SIS)**
SIS_HandFunction_	5.7 ± 16.4	2.9 ± 1.9	Severity, gender	0.134	0.139
	(5.7 ± 13.9)	(2 ± 1.1)		(0.180)	(0.07)
SIS_M__obility_	8.7 ± 9.8	4.4 ± 0.9	Severity, age, chronicity,	0.001***	0.00001***
	(7.2 ± 11.2)	(2.6 ± 0.7)	gender	(0.010)**	(0.00009)***
SIS_ADL_	5.9 ± 10.1	3.1 ± 0.2	Severity, concordance, age,	0.041*	0.0086**
	(4.9 ± 9.6)	(1.7 ± 0.8)	gender	(0.035)*	(0.054)*
SIS_Strength_	7.4 ± 13.9	3.7 ± 1.6	Severity, chronicity, gender	0.024*	0.021*
	(11.3 ± 12.l)	(1.7 ± 0.8)		(0.001)***	(0.00039)***
**Grip strength**	3.8 ± 8.1	1.9 ± 0.9	Severity, chronicity,	0.046*	0.037*
	(2.1 ± 7.7)	(1.0 ± 0.6)	concordance	(0.246)	(0.062)
**9-HPT_Affect_**	–5.9 ± 8.9	–2.9 ± 1.2	Chronicity	0.0081**	0.0201*
	(–4.5 ± 5.3)	(–1.9 ± 0.7)		(0.046)*	(0.0118)**
**Action research arm test (ARAT)**
ARAT_Total_	1.3 ± 2.4	0.6 ± 0.3	Severity, gender, chronicity,	0.046*	0.275
	(3.3 ± 4.9)	(1.1 ± 0.3)	gender	(0.020)*	(0.001)***
ARAT_Grip_	0.1 ± 0.5	0.03 ± 0.1	Severity, gender,	0.582	0.802
	(0.9 ± 1.4)	(0.3 ± 0.1)	concordance, chronicity, age	(0.025)*	(0.0059)**
ARAT_Grasp_	0.8 ± 1.6	0.4 ± 0.3	Severity, gender,	0.106	0.129
	(1.5 ± 3.6)	(0.5 ± 0.2)	concordance, chronicity,	(0.163)	(0.03)*
ARAT_Pitch_	0.4 ± 1.6	0.2 ± 0.2	age Seventy, gender,	0.289	0.215
	(0.6 ± 1.5)	(0.2 ± 0.1)	concordance	(0.106)	(0.039)*
ARAT_Gross_	0 ± 1.6	0 ± 0.02	Severity, age, chronicity,	1.00	1.00
	(0.3 ± 1.4)	(0.1 ± 0.1)	concordance, gender	(0.453)	(0.437)


*Scores, Covariates, and *p*-values are reported for *n* = 21 participants during BCI intervention: Mean improvement scores between time points 1 and 3, (parentheses) indicate mean improvement scores between time points 1 and 4. The time LME *p*-value is a *p*-value for the likelihood test between two models differing only in the inclusion of time as a covariate. ^∗^*p* < 0.05, ^∗∗^*p* < 0.01, ^∗∗∗^*p* < 0.00l.*

**Table 3:** Summary of Outcome Measures During Assessment and Including Follow-Up of BCI Therapy for Intervention vs. Control

Outcome measures	Control Improvement score Mean ± SD	Intervention Improvement score Mean ± SD	LME Estimate ± SE	Covariates	*T*-test *p-*value	Interaction LME *p*-value
**Stroke impact scale (SIS)**					
SIS_HandFunction_	0.4 ± 10.6	3.8 ± 10.8	2.6 ± 3.3	Severity, age, time, type	0.419	0.407
	(–0.9 ± 18.0)	(5.6 ± 7.3)	(2.1 ± 1.9)		(0.180)	(0.278)
SIS_M__obility_	5.1 ± 9.2	11.7 ± 12.0	1.8 ± 1.6	Seventy, chronicity, age,	0.197	0.237
	(2.7 ± 8.1)	(8.6 ± 13.1)	(1.5 ± 1.1)	gender, concordance, time, type	(0.085)	(0.148)
SIS_ADL_	3.5 ± 12.5	9.2 ± 13.4	1.2 ± 2.1	Severity, concordance,	0.397	0.567
	(0.2 ± 12.4)	(5.0 ± 10. 3)	(1.8 ± 1.3)	chronicity, gender, age, time, type	(0.156)	(0.175)
SIS_Strength_	2.6 ± 17.1	12.5 ± 8.8	2.4 ± 2.8	Severity, chronicity, gender,	0.149	0.379
	(4.1 ± 18.3)	(14.6 ± 10.3)	(4.4 ± 1.7)	time, type	*(0.019)	**(0.012)
**Grip strength**	–0.3 ± 6.4	1.7 ± 5.0	2.1 ± 1.5	Severity, age, time, type	0.526	0.163
	(3.4 ± 11.0)	(1.3 ± 3.6)	(–0.3 ± 0.9)	(chronicity)	(0.749)	(0.792)
**9-HPT_Affected_**	–7.7 ± 12.4	–2.6 ± 4.8	0.9 ± 2.8	Time, type (chronicity)	0.826	0.741
	(–2.5 ± 19.2)	(–3.8 ± 5.41)	(–0.8 ± 1.81)		(0.183)	(0.640)
**Action research arm test (ARAT)**					
ARAT_Total_	3.1 ± 4.08	0.4 ± 2.1	–0.8 ± 0.6	Severity, gender, age,	0.228	0.154
	(1.8 ± 3.8)	(3.2 ± 5.5)	(0.5 ± 0.5)	chronicity, time, type, concordance	(0.699)	(0.256)
ARAT_Grip_	0.3 ± 6.5	0.2 ± 0.4	–0.5 ± 0.3	Severity, gender, age,	0.514	0.075
	(3.4 ± 11.0)	(1.2 ± 1.6)	(0.1 ± 0.2)	concordance, chronicity, time, type	(0.195)	(0.458)
ARAT_Grasp_	1.1 ± 2.1	1.2 ± 1.9	0.03 ± 0.4	Severity, gender, age,	0.579	0.949
	(0.1 ± 0.4)	(1.0 ± 3.5)	(0.5 ± 0.3)	concordance, chronicity, time, type	(1.00)	(0.146)
ARAT_Pitch_	0.8 ± 2.1	–0.2 ± 0.4	–0.2 ± 0.3	Seventy, concordance,	0.391	0.508
	(0.3 ± 2.1)	(0.3 ± 0.8)	(0.2 ± 0.2)	time, age, gender, type (chronicity)	(0.704)	(0.501)
AKAT_Gross_	0.3 ± 0.5	1.0 ± 1.9	–0.2 ± 0.3	Severity, age, concordance,	1.00	0.46
	(0.8 ± 1.2)	(0.2 ± 0.5)	(–0.2 ± 0.2)	chronicity, time, type (gender)	0.252	(0.303)


*Scores, covariates, and *p*-values are reported for *n* = 21 participants during BCI intervention: mean improvement scores between time points 1 and 3, (parentheses) indicates mean improvement scores between time points 1 and 4. Interaction LME *p*-value is a *p*-value for the likelihood ratio test between two LME models differing only in the inclusion of a time:type interaction term (where type is either intervention period or control) as a covariate. ^∗^*p* < 0.05, ^∗∗^*p* < 0.01, ^∗∗∗^*p* < 0.001.*

**Table 4:** Summary of outcome measures during assessment and including follow-up for responders vs. non-responder.

Outcome Measures	Responder improvement score Mean ± SD	Non-responder improvement score Mean ± SD	LME Estimate ± SE		Covariates	*T*-test *p-*value	Interaction LME *p*-value	Response LME *p-*value
**Stroke impact scale (SIS)**								
SIS_HandFunction_	4.4 ± 21.4	10.0 ± 11.8	–2.8 ± 4.2	6 ± 5.9	Severity, *time, response*	0.901	0.498	0.26
	(7.7 ± 19.54)	(3.0 ± 4.5)	(0.4 ± 2.3)	(7 ± 5.6)	(chronicity)	(0.544)	(0.877)	(0.179)
SIS_M__obility_	9.2 ± 8.0	12.8 ± 13.4	–1.3 ± 2.3	19.6 ± 5.8	Seventy, age, chronicity,	0.382	0.564	0.000213^∗∗∗^
	(8.0 ± 8.6)	(11.6 ± 18.9)	(–1.4 ± 1.8)	(18.6 ± 6.9)	concordance, gender, *time, response*	(0.487)	(0.405)	(0.00155)^∗∗^
SIS_ADL_	8.6 ± 11.0	10.0 ± 10.8	–0.7 ± 3.1	10 ± 6.8	Seventy, concordance,	0.295	0.819	0.0515
	(6.3 ± 9.8)	(9.5 ± 9.6)	(–2.0 ± 2.0)	(9.7 ± 7.4)	gender, age, *time*, *response*	(0.523)	(0.291)	(0.0795)
SIS_Strength_	6.2 ± 13.6	2.5 ± 15.7	1.9 ± 4.4	15.4 ± 9.7	Severity, gender, *unit*,	0.255	0.661	0.049^∗^
	(11.8 ± 11.0)	(10.0 ± 16.9)	(0.04 ± 2.8)	(14.8 ± 9.2)	*response*	(0.430)	(0.988)	(0.048)^∗^
**Grip Strength**	3.0 ± 7.4	1.0 ± 2.2	1.0 ± 2.1	6.1 ± 3.7	Seventy, chronicity,	0.399	0.617	0.082
	(1.7 ± 11.0)	(1.3 ± 2.3)	(0.4 ± 1.2)	(5.5 ± 3.5)	concordance, *time, response*	(0.864)	(0.766)	(0.095)
**9-HPT Affected**	–	–	–	–	–	–	–	–
**Action research arm test (ARAT)**
ARAT_Total_	3.5 ± 5.2	–0.8 ± 1.3	1.6 ± 0.5	4.4 ± 4.8	Severity, gender, age,	0.242	0.0026**	0.239
	(4.4 ± 6.2)	(1.4 ± 1.9)	(1.2 ± 0.6)	(5.1 ± 5.3)	concordance, chronicity, *time*, *response*	(0.204)	(0.078)	(0.214)
ARAT_Grip_	0.2 ± 0.4	–0.2 ± 0.4	0.2 ± 0.2	0.9 ± 1.1	Severity, gender, chronicity,	0.399	0.212	0.226
	(1.3 ± 1.4)	(0.4 ± 1.5)	(0.3 ± 0.2)	(1.2 ± 1)	concordance, *time, response*	(0.208)	(0.158)	(0.099)
ARAT_Grasp_	1.6 ± 1.8	–0.2 ± 0.4	0.9 ± 0.5	1.9 ± 2	*Time*, *response* (age,	0.347	0.09	0.236
	(2.4 ± 4.6)	(0.4 ± 1.5)	(0.6 ± 0.4)	(2.4 ± 1.7)	chronicity)	(0.495)	(0.151)	(0.159)
ARAT_Pitch_	0.7 ± 2.1	0 ± 0	0.04 ± 0.04	1.0 ± 1.1	Severity, chronicity, *time*,	1.0	0.322	0.296
	(1.1 ± 1.8)	(0.3 ± 2.1)	(0.4 ± 0.2)	(1.1 ± 1.1)	*response*	(0.907)	(0.067)	(0.236)
AKAT_Gross_	0.2 ± 1.5	0.4 ± 1.8	0.3 ± 0.4	0.8 ± 1.3	Severity, age, concordance,	0.621	0.463	0.43
	(0.1 ± 1.6)	(0.6 ± 0.9)	(–0.1 ± 0.3)	(0.4 ± 1.4)	gender, *time*, *response* (chronicity)	0.864	(0.727)	(0.509)


*Scores, covariates, and *p-*values are reported for *n* = 21 participants during BCI intervention: mean improvement scores between time points 1 and 3, (parentheses) indicate mean improvement scores between time points 1 and 4. Interaction LME *p*-value is a *p*-value for the likelihood ratio test between two LME models differing only in the inclusion of a time:response interaction term as a covariate. Response LME *p*-value is a *p*-value for the likelihood ratio test between two LME models differing only in the inclusion of a response term as a covariate with neither models including an interaction term. ^∗^*p* < 0.05, ^∗∗^*p* < 0.0l, and ^∗∗∗^*p* < 0.001.*

Outcome measures used in all analyses included ARAT, Hand Grip Strength, and the 9HPT as well as SIS measures of Hand Function, Mobility, ADLs, and Strength of the hemiparetic side. For each analysis, and for each outcome measure utilized, ceiling scores (participants who recorded a maximum outcome score at baseline and completion for ARAT) were removed given the impossibility for measured improvement. On the other hand, floor scores (participant data that demonstrated a minimum outcome score at the intervention baseline measure) remained in all analyses akin to an intent-to-treat standard. Given this selection, the sample size across all data remained at *n* = 21 and *n* = 14 for the responder sub-analyses for most outcome measures. The outcome measurements with sample size adjustments following the above criteria include ARAT (*n* = 14 for both analyses) and SIS_hf_ (*n* = 20). Additionally, the sample size of 9HPT_affected_ (*n* = 9 overall, *n* = 2 in the responder dichotomization) was greatly reduced from the original sample of 21 due to participants’ inability to complete the task given the extent and severity of their UE impairment.

Independent samples *t-*tests utilized only DTG control data and ITG intervention data (neglecting the use of DTG intervention data) so as not to introduce an inter-subject dependence of the analyses. Meanwhile, the LME analyses used a random effect for subjects to account for the non-independence of the longitudinal data and used all subject time points. For each mixed model testing a specific outcome, relevant covariates to control for were chosen based on stepwise regression analysis. For each outcome measure with the selected covariates, two nearly identical mixed models were created that differed only in the inclusion of a single covariate of interest. When examining how subjects’ outcome scores changed with time, the covariate of interest was the time period (1, 2, 3, or 4) of interventional assessment. For comparing the intervention to control, both LME models included the independent effects of time and therapy type (control or intervention) and stringently tested for improvement rate differences by inclusion of an interaction term between time and type as the covariate of interest. Similarly, both models in the responder sub-analyses included independent effects of time and response (responder or non-responder) and stringently tested for improvement rate changes through an interaction term between time and response. Meanwhile, response was used as the covariate of interest to test if responders showed general differences in secondary outcome measures compared to non-responders. Finally, a similarly run generalized linear model (GLM) analysis examined potential significant covariates that helped predict whether a subject would become a responder through this BCI intervention. The specific covariates tested included stroke severity, chronicity, and concordance, as well as age, gender, and baseline ARAT scores. All mixed modeling analyses were completed in RStudio (Version 0.99.903 – 2009–2016 RStudio, Inc.). The *t*-tests were run using SPSS (Version 22). Thresholds for significance were set *a priori* at *p* ≤ 0.05 for all statistical analyses.

### *Post Hoc* Rational: Dichotomizing Responders

Two groups, deemed “responders” and “non-responders” (Snapinn and Jiang, 2007), were generated *post hoc* from this sample based on whether positive change in the primary objective measure of UE function was realized following BCI intervention (completion assessment score – baseline assessment score). The grouping of responders vs. non-responders is represented in Tables 1 and 5. Table 1, the main demographics table, denotes responders with asterisks in the completion ARAT score column. Table 5 demonstrates relevant summary characteristic differences between the dichotomized groups.

**Table 5:** Demographic distribution by ARAT score response.

Response	Participants	Age (years) mean ± SD	Females (males)	Acute (chronic)	Mild (severe)	Concordant (non-concordant)
Responder	9	62.6 ± 14.3	5 (4)	1 (8)	2 (7)	2 (7)
Non-responder	5	58.3 ± 12.9	3 (2)	2 (3)	0 (5)	0 (5)
Total	14	61.1 ± 13.5	8 (6)	3 (11)	2 (12)	2 (12)


*Concordant strokes are classified as those predominantly affecting the preferred arm as assessed by the Edinburgh Handedness Inventory [30]. Individual responder and non-responder demographics are highlighted on ARAT outcome denoting the responders.*

### The BCI System

#### BCI Software and EEG Hardware

The BCI system and intervention sequence were consistent with those previously described (Wilson et al., 2012; Song et al., 2014, 2015; Young et al., 2014a,b,c,d, 2015), using BCI 2000 software (Schalk et al., 2004) version 2 with in-house modifications for input from a 16-channel EEG cap and amplifier (Guger Technologies) and integration with tongue stimulation (TDU) (TDU 01.30 Wicab Inc.) (Kaczmarek, 2011) and functional electrical stimulation (FES) of distal UE muscles (LG-7500, LGMedSupply; Arduino 1.0.4) associated with grasping behavior.

#### Functional Electrical Stimulation

Functional electrical stimulation of the UE was delivered using the LG-7500 Digital Muscle Stimulator (LGMedSupply, Cherry Hill, NJ, United States). Stimulus was conducted through a pair of 2” × 2” square electrodes placed securely on the affected forearm using highly conductive Electrolyte Spray. The electrodes were placed to facilitate either a grasping motion (finger flexion), or finger extension according to participant preference. Specific placement sites were superficial to digitorum superficialis to facilitate hand and finger flexion, or superficial to extensor digitorum communis to facilitate hand and finger extension. The natural absence of a flexor digitorum superficialis tendon to the fifth digit in some individuals was not considered by this study design. Stimulation was controlled through the PC using an Arduino Uno R3 (Adafruit Industries, New York, NY, United States) and a simple reed relay circuit, with the amplitude set to elicit observable muscle activation (e.g., finger grasping) without pain. The pulse rate of the stimulation was set to 60 Hz to produce tetanic contraction of the muscle; the pulse width was set to 150 μs. The input signal, initially set to zero, was adjusted by steps of 0.5 mA, unless the stimulation became uncomfortable for the subject. The device was never set to deliver an output greater than 5.0 mA.

#### Tongue Display Unit

In previous publications, the TDU has been described and its use in a BCI paradigm detailed (Schalk et al., 2004; Kaczmarek, 2011; Wilson et al., 2012). This BCI system uses the same TDU stimulation parameters as were previously reported (Wilson et al., 2012).

### BCI Intervention Procedure

#### Familiarization With the BCI Device and Procedures

The first BCI session was focused on assisting the participant to comprehend and engage the BCI device and protocol. Stroke survivors often present with a myriad of cognitive, affective, and physical impairments (Nair et al., 2015; Stinear, 2016) and out of respect for individual participant needs and abilities, the researchers provide at outset an opportunity for a generous orientation rather than rigorous acquisition. During this preliminary session, the EEG cap, FES device, and TDU device were faithfully administered as described previously (Wilson et al., 2012). Participants were instructed before each session, and as needed, to aim for successful completion of BCI tasks and for each attempted movement to be performed to the participant’s autonomously elected level of comfort, ability, and pleasure. The proposed design entails at least 10 runs for each closed-loop condition per session; however, enforcement discretion was encouraged until a participant demonstrated task comprehension.

#### Cursor Task and User Integration

In the closed-loop BCI intervention task, participants perform attempted actual hand movements in response to a left or right target cue displayed on a computer screen as a virtual *ball-and-target* (Young et al., 2015; Figure 2). To accommodate initial movement capacity and recovery goals, best possible attempts at repeated hand grasping (finger extension and flexion) were used. Participants learn to control horizontal movement of a virtual ball displayed on the monitor by modulating their sensorimotor rhythm (SMR) activity (SMR activity represents Mu and Beta rhythm changes over the motor cortex – this process is indicative of healthy normal brain electrophysiology of attempted movement) as they perform the task (Wilson et al., 2012). The SMR activity related to attempted left (or right) hand movements, as recorded by the EEG, is then translated into leftward (or rightward) ball movement via the BCI (Wilson et al., 2012). Mu and beta SMRs in human subjects (Muralidharan et al., 2011) are recorded exclusively over sensorimotor areas at frequencies of about 8–12 and 16–24 Hz (Pfurtscheller et al., 1997; Riehle and Vaadia, 2004; Birbaumer et al., 2006), with the source of human SMR in the sensorimotor regions following the homuncular organization of the motor and somatosensory cortical strip (Pfurtscheller et al., 1997; Riehle and Vaadia, 2004). At the start of each intervention trial, a virtual target randomly appears on the left or right side of the screen. After 1 s, a virtual ball appears in the center of the screen, and the subject is instructed to move the ball toward the target by eliciting SMR modulation using attempted hand movement. For a trial to be considered successful, the ball must hit the target within 5 s of its appearance. Trials are aborted and considered unsuccessful if, after 5 s, the ball has not reached the target. The inter-trial interval is 3 s regardless of aborted or successful trial (Figure 2).

#### Adjuvant Stimulus Schedule

Following completion of at least 10 runs of the visual only BCI task described above, adjuvant FES stimulation was applied to the muscles of the impaired hand, and electro-tactile feedback (visual replication and supplementation) was presented when available through the TDU for the duration of the trials possible in a 2-h session. In this way, subjects might utilize visual feedback, muscle stimulation, and electro-tactile feedback (or visual replacement or supplementation in the case of uncorrected visual impairment) to monitor their task performance. FES-driven stimulation, however, was only applied to the impaired limb and concordant with both ball movement toward the impaired side, and the virtual target presenting on the impaired side. In this way, externally facilitated muscle stimulation never occurred while the subject was attempting to move the ball toward their unimpaired side.

## Results

### Primary Effect of BCI Intervention

Of the *n =* 21 participants, 14 participants had room for improvement in the ARAT of which 64% (9/14) realized improved scores in the primary outcome measure (ARAT_total_) from baseline to completion of intervention, both at immediate completion and/or 1-month post-completion (Table 1). 43% (6/14) had changes in the ARAT that are considered to meet significant ARAT specific thresholds [four of these participants had MDC ≥ 3 (MDC_90_ = 3.0; Simpson and Eng, 2013) and two of these participants had MCID ≥ 5.7 both at immediate completion and/or 1-month post-completion]. The seven participants who had no room for improvement, or had a max score of 57 at ARAT, stayed at the same max level in ARAT both at immediate completion and 1-month post-completion.

#### Effect of Intervention Time on Outcome Scores

A paired samples *t-*test found a significant effect of time on ARAT outcome improvement score (*p* = 0.046). Secondary outcome measures found to have significant effect over time included SIS_mobility_ (*p* = 0.001), SIS_adl_ (*p* = 0.041), SIS_strength_ (*p* = 0.024), as well as Hand Grip Strength (*p* = 0.046) and 9HPT_affected_ (*p* = 0.0081) (Table 2).

Likelihood ratio tests of LME models over time periods 1–3 controlling for severity, gender, chronicity, and concordance did demonstrate a significant effect of time on ARAT outcome score improvement (*p* = 0.02754) (Table 2). Specifically, the full LME model revealed an estimate improvement rate of ARAT score by 0.64 ± 0.28 (μ ± SE) between time periods. In addition, the LME model found significance for the secondary outcome measures of SIS_mobility_ (*p* = 0.00001), SIS_adl_ (*p* = 0.008613), SIS_strength_ (*p* = 0.0212), Hand Grip Strength (*p* = 0.0368), and 9HPT_affected_ (*p* = 0.0201) while controlling for the most significant covariates as determined by forward stepwise regression (Table 2).

#### Including Follow-Up

A paired samples *t*-test evaluated between baseline and follow-up demonstrated a significant effect of ARAT improvement score (*p* = 0.020). Many secondary measurements at follow-up demonstrated similarly significant improvements including SIS_mobility_ (*p* = 0.010), SIS_adl_ (*p* = 0.035), SIS_strength_ (*p* = 0.001), and 9HPT_affected_ (*p* = 0.046) (Table 2).

The likelihood ratio tests of the LME models across follow-up also demonstrated significant improvement in ARAT, controlling for severity, gender, chronicity, and concordance (*p* = 0.0010) (Table 2). The estimated improvement rate of ARAT score was 1.06 ± 0.31 (μ ± SE) between time periods. The likelihood ratio tests also revealed significance among SIS_mobility_ (*p* = 0.00009), SIS_strength_ (*p* = 0.00039), and 9HPT_affected_ (*p* = 0.01178) (Table 2).

### ARAT Improvement Rate Between Control and Intervention (Therapy Type)

#### During Assessment Period

When testing between Control (*n* = 12) vs. Intervention (*n* = 9) therapy types, the independent samples *t*-tests did not find that subjects during intervention improved in ARAT outcome score at a significantly faster rate than controls. Additional measures via *t*-tests found no significant differences between control and intervention from time points 1–3 (Table 3).

A likelihood ratio test controlling for severity, gender, age, chronicity, concordance, and the independent effects of time and therapy type (control or intervention) also did not find a significant effect of the specific interaction term between time and therapy type for ARAT outcome score (*p* = 0.1543) (Table 3). Similarly, improvement rates for secondary measurement outcome scores between intervention and control from time points 1–3 were not significant while controlling with forward stepwise regression selected covariates and the independent effects of time and therapy type (Table 3).

#### Including Follow-Up

**FIGURE 3:**
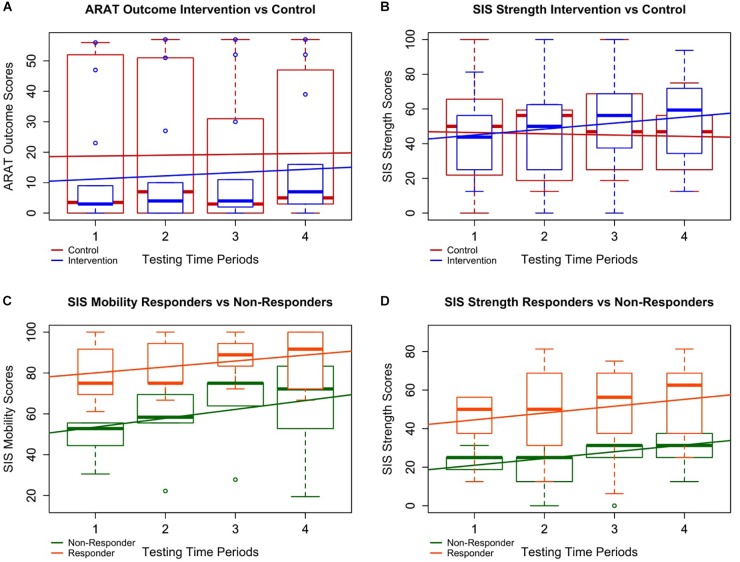
Intervention vs. control and responder vs. non-responder plots. Four of the most notably significant relationships are plotted with boxplots of all patient data overlaid by simple linear best fit lines to depict general trends in the data. **A** and **B** specifically demonstrate differences in the data between all controls (in red) and all interventions (in blue) whereas **C** and **D** represent trends in the data between responders (in orange) and non-responders (in green). **(A)** Although the improvement rate in ARAT for subjects in intervention was not significantly higher than controls, participants in intervention did significantly improve over time, and the trend of the boxplot medians suggests a possible continuation of improvement through follow-up not present in the control period. **(B)** Participants in intervention significantly improved faster over time in SIS_strength_ than those in the control period despite both groups starting at similar levels of ability. **(C, D)** Responders demonstrated significantly higher average SIS_mobility_ and SIS_strength_ scores than non-responders. This suggests patients with lower SIS_mobility_ and SIS_strength_ scores may not benefit from BCI intervention as well as those with higher scores.

The *t*-test assessed at follow-up did not find a significant effect of ARAT outcome improvement score. However, there was a significant effect of SIS_strength_ improvement score (*p* = 0.019) (Table 3). The likelihood ratio tests at follow-up for ARAT, controlling for severity, gender, age, chronicity, concordance, and the independent effects of time and type were not significant (*p* = 0.256) (Table 3 and Figure 3A). Like the *t*-test, there was a significant effect between control and intervention for SIS_strength_ (*p* = 0.0117) when controlling for severity, chronicity, gender, and the independent effects of time and therapy type (Table 3 and Figure 3B).

### ARAT Improvement Rate Between Responders and Non-responders (Response Type)

#### During Assessment Period

When testing between responders (*n* = 9) vs. non-responders (*n* = 5), neither *t*-tests nor likelihood ratio tests of generalized mixed effect models found the individual covariates of age, gender, chronicity, severity, concordance of strokes, or baseline ARAT scores to significantly predict a subject’s ability to improve in ARAT outcome over the course of intervention. LME analyses demonstrated that, while controlling for severity, gender, chronicity, concordance, and the independent effects of time and response, responders improved significantly faster than non-responders by 1.62 ± 0.51 (μ ± SE) points per time point through intervention (Table 4). LME analyses further revealed significant positive differences between responders and non-responders in SIS_mobility_ by intervention completion (*p* = 0.0002) and SIS_strength_ (*p* = 0.04995) (Table 4 and Figure 3C). Specifically, responders demonstrated increased SIS_mobility_ scores of 19.63 ± 5.75 (μ ± SE) and increased SIS_strength_ scores of 15.38 ± 9.67 through intervention.

#### Including Follow-Up

When testing between responders (*n* = 9) vs. non-responders (*n* = 12), neither *t*-tests nor likelihood ratio tests of generalized mixed effect models found the individual covariates of age, gender, chronicity, severity, concordance of strokes, or baseline ARAT scores to significantly predict a subject’s ability to improve in ARAT outcome through follow-up. LME analyses did not demonstrate a significant difference in improvement rates in ARAT between responders and non-responders through follow-up while controlling for severity, gender, chronicity, concordance, and the independent effects of time and response (*p* = 0.07821) (Table 4). However, LME analyses did reveal significant positive differences between responders and non-responders in SIS_mobility_ (*p* = 0.00155) and SIS_strength_ (*p* = 0.04828) through follow-up while controlling for the forward-step selected covariates (Table 4 and Figure 3C). Specifically, responders demonstrated increased SIS_mobility_ and SIS_strength_ scores of 18.59 ± 6.88 and 14.80 ± 9.23 (μ ± SE), respectively, through follow-up while controlling for the selected covariates (Table 4 and Figure 3D).

### Identifying Patients for BCI Intervention

These data suggest that particular participant characteristics may be associated with greater gains of functional capacity. The covariates of severity, concordance of strokes, age, gender, and chronicity, within this limited sample size, may not, at this sampling, significantly predict whether a participant will improve in ARAT primary outcome scores due to BCI intervention. However, increased SIS_mobility_ and SIS_strength_ scores do significantly help predict response outcome (Table 4). It is further possible that other outcome scores relatively close to significance (*p* ≤ 0.1), such as SIS_adl_ and Hand Grip Strength (Table 4), may prove significant with an increase in sample size. Additionally, although gender, chronicity, severity, or concordance did not significantly predict if a participant would become a responder, 73% (8/11) of chronic and 100% (2/2) of mild participants who had room for ARAT improvement became responders. Responders to this intervention schedule were, like the larger cohort sample, a heterogeneous group and included survivors with severe motor impairment of non-dominant hand (Table 5) as measured post stroke. It may be possible to extrapolate upon these data, strengthened by systematic review of existing literature, to identify patients prepared to realize optimal recovery outcomes with BCI intervention.

## Discussion

### Prescribing BCI as UE Therapy

Brain–computer interface intervention can impact functional motor capacities of the impaired UE (Remsik et al., 2016), and in this sample, primary outcome measurements of distal UE function did significantly improve from baseline to completion as well as baseline to follow-up (Table 2). Results also suggest the delayed therapy condition utilized in this cross-over controlled design did not adversely affect UE impairments in individuals randomized into the DTG. Participants in intervention showed greater rate of change compared to control (Figure 3A) as well as greater average gains by completion. However, these differences were not statistically significant. Insufficient power, especially following the removal of ceilings, as well as the duration of specific neural plastic changes (weeks, months, or longer) (Jones, 2017), may contribute to this lack of significant differences.

Although BCI intervention appears to lead to functional reorganization of the central nervous system, or brain (Caria et al., 2011; Song et al., 2014, 2015; Zich et al., 2017; Cervera et al., 2018), it is not unreasonable to suggest that more time in therapy is needed for these CNS changes to manifest as measurable, clinically relevant changes in UE behavior. This possibility may explain the delay in primary outcome improvement between baseline and midpoint medians (2–3 weeks apart) compared to the differences between baseline and completion or even the middle time point and completion (Figure 3A). This assumption is supported by the continued improvement between midpoint and follow-up for those in intervention, a change which is not observed in the control group (Figure 3A). This delay of 2–3 weeks of the larger primary outcome score change is also consistent with a similar BCI therapy research design (Li et al., 2014). Further analysis about the rate of change at various time points is needed.

Mean projected FMA-UE changes from baseline to follow-up in this sample (5.4) are comparable to improvements in FMA-UE baseline to completion score changes (Cervera et al., 2018) in other published experimental BCI intervention studies. Subchronic patients generally experience greater therapeutic effects of BCI interventions than do chronic participants (Cervera et al., 2018), and a similar limiting relationship may exist between mild and severe UE impairment patients (Cramer and Nudo, 2010; Stinear and Byblow, 2014). Such trends may account for some differences between the presented projected FMA-UE score changes estimated from this sample (mean change of 2.2 and 5.4 at completion and 1-month post-completion, respectively) (Table 1), which are potentially labored by the heterogeneity of time since stroke and level of physical impairment post-stroke, and greater changes reported in similar studies (Li et al., 2014; Kim et al., 2016) by other groups. For example, Li et al. (2014) (*n* = 7) demonstrated a 12.7 FMA-UE change, however with a sample of subjects that was much less chronic (all chronicity ≤ 6 months) than those participants examined herein (Li et al., 2014). Similarly, Kim et al. (2016) (*n* = 15) saw a 7.87 change in FMA-UE scores, however on average (baseline μ_FMA-UE_ = 26.8), those subjects had less severe strokes (Kim et al., 2016) than the participants in this sample. In general, most BCI intervention studies remain underpowered and inadequately constrained (Cervera et al., 2018), presenting threats to both internal and external validity.

The results of this study suggest that SIS_mobility_ and SIS_strength_ may be important factors to consider when designing or prescribing BCI regimes as higher scores were significantly indicative of increased likelihood for treatment success. While still unclear, other factors that may also play predictive roles in BCI interventional motor recovery include, but are not limited to, Hand Grip Strength and SIS_adl_ scores, as well as stroke chronicity and severity. While insignificant due to the small sample size, the large proportions of chronic and mild patients who became responders, 73% (8/11) and 100% (2/2), respectively, does follow previously reported trends (Caria et al., 2011; Ang and Guan, 2013; Young et al., 2014d; Ang et al., 2015; Remsik et al., 2016). The fact that BCI intervention appears to be able to specifically benefit chronic patients is especially interesting as many stroke patients reach a functional recovery plateau by completion of standard of care treatment (Wolf et al., 2006, 2010; Dromerick et al., 2009; Cramer and Nudo, 2010). The heterogeneity of these data and relatively small sample size may limit the external validity of all reported trends as well as limit the realization of other important predictors.

To date, the literature exploring the behavioral and rehabilitative implications of BCI treatments remains underpowered. Nonetheless, this body of research has shown rapid growth in the last decade and a half (Ang and Guan, 2013; Remsik et al., 2016; Bundy et al., 2017; Cervera et al., 2018). Research assessing which presenting stroke patients will profit most from BCI treatments remains mostly inconclusive. However, increased microstructural integrity of the ipsilesional posterior limb of the internal capsule (PLIC) has been correlated with greater motor recovery from BCI therapy (Song et al., 2014, 2015). Similarly, Young et al. (2016) demonstrated that changes to the integrity of the contralesional corticospinal tract (CST) during BCI therapy correlates to behavioral improvement scores for ARAT and 9-HPT. Thus far, most BCI treatment studies have observed participants in the chronic stage of stroke. As BCI is still a relatively new concept for treatment of UE paresis, it is possible that the majority of individuals participating in BCI research have exhausted standard clinical care. Thus, samples may be weighted disproportionately by participants with chronic persistent UE motor disability. It is also possible that the therapeutic impact of BCI intervention is dependent on several factors (i.e., residual motor capacity, lesion volume, and time since stroke) which should be considered before BCI treatment is prescribed (Stinear and Byblow, 2014). A forthcoming intent to treat analysis of this study should help address some of these unanswered questions in a more robust manner.

### Motivational Influences of BCI Use

Changes in primary outcome scores (ARAT) during treatments suggested that this BCI design may deliver moderate objective positive UE motor changes, as seen in the 64% (9/14) of participant (out of those who had room for improvement) “Responders” who completed the BCI treatments protocol as designed. 43% (6/14) had changes in the ARAT who are considered to meet significant ARAT-specific thresholds [four of these participants had MDC of at least 3 (MDC_90_ = 3.0; Simpson and Eng, 2013) and two of these participants had MCID of at least 5.7 both at immediate completion and/or 1-month post-completion]. Additionally, the largest positive changes compared to baseline in ARAT were observed 1-month post treatment for a few participants. This might suggest that continuation of biological and behavioral recovery mechanisms induced by BCI systems may remain active in participants beyond their time in the lab setting.

### Limitations

#### Suitability of Dichotomized Responder Analysis as a Sufficient Measure of Clinical Importance of Treatment Effects

A significant portion of this publication is dedicated to an analysis of participants according to *post hoc* dichotomized assignment by main effect in the primary outcome. Responder analyses are challenged by several inherent limitations (Snapinn and Jiang, 2007). First, the arbitrariness of a “responder” threshold value levies a substantial cost as dichotomization decreases efficiency and increases sample size requirements (limited power relative to analysis of the original selection). Further, the motivation for a responder analysis is to assess clinical relevance (to ensure clinical relevance of treatment effect), and as clinical relevance is ubiquitous with every clinical trial and setting, such logic may be seen as inherently circuitous. Beyond the inherent shortcomings of a *post hoc* responder analysis, this study was constrained by heterogeneity in many covariates including lesion location, level of impairment, age, gender, and time since stroke among the participants studied. Certainly, greater power is needed to adequately generalize results to a more adequate standard.

#### Nature of the Academic Research Environment

This is an ongoing study in its seventh year of data acquisition and enrollment. Multiple different project personnel have undergone and supervised the staffing, training, and data acquisition of this trial during its course. The authors work hard to best minimize differences in acquisition of study measures through extensive and repeated training of personnel.

## Conclusion

Both primary (ARAT) and secondary (SIS_mobility_, SIS_adl_, SIS_strength_, Hand Grip Strength, and 9HPT_affected_) outcome measures were significantly improved over the course of this BCI interventional therapy. For SIS_strength_ scores specifically, participants in intervention demonstrated significantly increased improvement rates through follow-up compared to controls while controlling for severity, chronicity, gender, and the independent effects of time and therapy type as measured through likelihood ratio tests of LME models. None of the analyses revealed any significant negative effect of delaying BCI treatments for participants. This particular result may be attributed to the chronicity of most of the recruited participants (*n* = 16 ≥ 1 year, *n =* 17 ≥ 6 months) since patients typically reach a functional plateau before the chronic phase of stroke and are not expected to realize a large degree of change, rehabilitative or otherwise, to their UE motor capacity. This particular study did not reveal significant differences between those who demonstrated improvement in ARAT outcome and those who did not in terms of age, gender, chronicity, severity, or concordance of stroke impairment suggesting that the BCI intervention design may be suitable for a large range of patients. However, 8/11 chronic, and both mild, participants with room for ARAT score improvement achieved “responder” designation, and the explicit capacity of BCI treatments to assist chronic (and mild) stroke patients, even after they have reached a functional plateau, is reported in other literature (Caria et al., 2011; Ang and Guan, 2013; Young et al., 2014d; Ang et al., 2015; Remsik et al., 2016). Despite statistical limitations of the heterogeneity of the relatively small sample size in this study, those who responded to the BCI intervention did have significantly higher self-reported SIS_mobility_ and the SIS_strength_ scores through follow-up. These findings may suggest that particular measures can assist in the prescription of a BCI intervention regimen necessary for an individual participant, as well as aid in the prediction and measurement of BCI interventional success as assessed by primary outcome measures of capacity and performance, like the ARAT.

Additional research is required to identify how BCI intervention dose–response relationships are influenced by the various potential classifications of stroke survivors. It is quite possible that prescribing BCI intervention as a one-size fits all treatment for UE motor impairment may not be an ideal approach for this rehabilitative technology. Rather, these data suggest that at least some outcome measures, along with stroke severity and chronicity, may prove valuable in determining if BCI treatments could be effective for a stroke survivor with persistent UE paresis. Therefore, patients receiving BCI treatments in future research or clinical contexts might benefit most from a treatment regimen tailored to the individual’s presenting performance capacity as measured by the easily administered and scored SIS. Supplementary outcome measures (both objective and self-reported), impairment characteristics, and treatment goals should all be taken into account when designing a BCI intervention for a potential participant. Future studies should seek to more thoroughly examine the effects of patient characteristics on BCI effectiveness, and examine how to deliver targeted treatments based on individual impairments and treatment goals in a concerted effort to maximize rehabilitative effect with similar BCI intervention strategies.

## Author Contributions

AR and KD were involved in subject recruitment, data collection, data processing, analyses, as well as interpreting and writing of the manuscript. LW was involved in preprocessing data, writing, and editing the manuscript. KG and CR were involved in interpreting and writing of the manuscript. JT, TJ, JA, MMc, and MM were involved in data collection, analyses, and editing the manuscript. BY was involved in subject recruitment, data collection, and manuscript editing. ZN was involved in subject recruitment and data collection. MW, MM, MMc, SR, HA, RM, NT, LW, and JA were involved in data collection. VN contributed to data collection, manuscript editing and intellectual content. PvK contributed to manuscript editing and intellectual content. TK was involved in the recruitment of study participants. JS was involved in recruitment of study participants and intellectual content. JW, VP, and DFE are co-PIs and were involved in study conception, design, manuscript editing, and intellectual content.

## Conflict of Interest Statement

The authors declare that the research was conducted in the absence of any commercial or financial relationships that could be construed as a potential conflict of interest.
