# Supplementary figures and images for: Behavioral Outcomes Following Brain–Computer Interface Intervention for Upper Extremity Rehabilitation in Stroke: A Randomized Controlled Trial
Source: Front Neurosci. 2018 Nov 8;12:752. doi: 10.3389/fnins.2018.00752 (PMC6235950; doi:10.3389/fnins.2018.00752)

## CONSORT Behavioral Flow Diagram

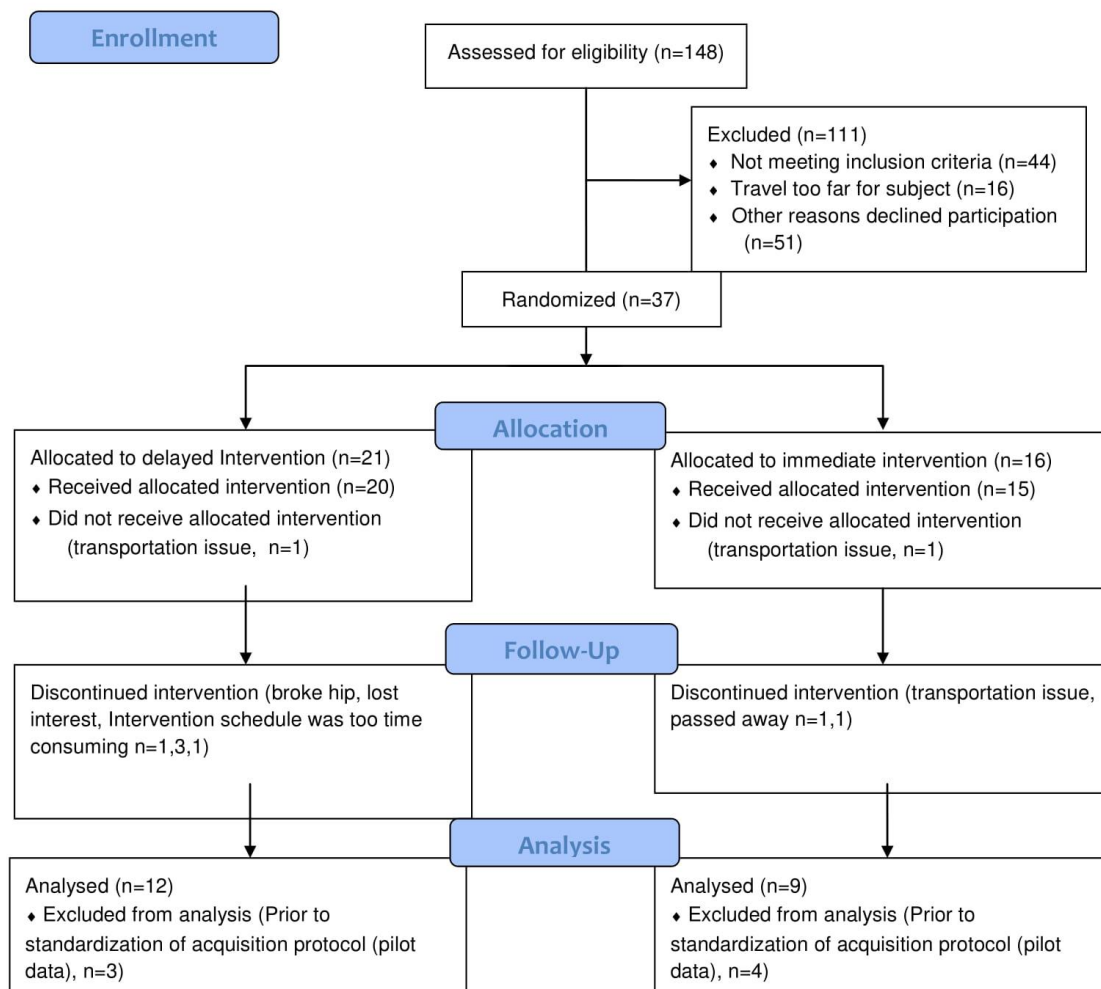

Supplement: Supplementary file 1 [file Data_Sheet_1.PDF]
